# Characterization of *Alternaria* species associated with Alternaria leaf blight of mustard reveals the genetic toxigenic potential of *Alternaria alternata*

**DOI:** 10.3389/fpls.2026.1901357

**Published:** 2026-09-07

**Authors:** Chetan Chopra, Shubha Trivedi, Partha Sarathi Tripathy, Prashant P. Jambhulkar, Anita Puyam, Rakesh Choudhary

**Affiliations:** 1 Department of Plant Pathology, Rani Lakshmi Bai Central Agricultural University, Jhansi, UP, India; 2 Department of Fisheries, Rani Lakshmi Bai Central Agricultural University, Jhansi, UP, India; 3 Department of Genetics and Plant Breeding, Rani Lakshmi Bai Central Agricultural University, Jhansi, UP, India

**Keywords:** *Alternaria alternata*, *Alternaria brassicae*, *Alternaria brassicicola*, leaf blight, mustard

## Abstract

*Alternaria leaf* blight (ALB), caused by the *Alternaria* species complex, is an economic threat to mustard cultivation all over the world. In this study, 700 infected leaf samples were collected from 28 blocks across six districts of the Bundelkhand region, Uttar Pradesh, India. To assess the prevalence and distribution of *Alternaria* species, 28 representative isolates were selected for further study. Morphological traits separated the majority of the Alternaria isolates into three morphotypes corresponding to *A. brassicae*, *A*. *brassicicola*, and *A*. *alternata*, which cause leaf blight. The pathogenicity test revealed that 18 isolates were highly virulent, 6 isolates were moderately virulent, and 4 were less virulent. Molecular analyses of the 18 highly virulent isolates, based on the internal transcribed spacer (ITS) region and the Alternaria-specific *Alt a1 marker*, confirmed the presence of *A. brassicae* (6 isolates), *A. brassicicola* (3 isolates), and *A. alternata* (9 isolates) in the region. Haplotype network analysis of concatenated ITS and Alt a1 sequences revealed two haplotypes among the *A. alternata* isolates and one haplotype each among the A*. brassicae* and *A. brassicicola* isolates. Additionally, all virulent A*. alternata* isolates carried the polyketide synthase genes (*pksH* and *pksJ*), indicating their genetic potential for Alternaria toxin biosynthesis. The predominance of *A*. *alternata* in mustard crops and the presence of toxin-biosynthesis-associated genes may pose a threat to edible oil production and safety, thereby raising serious concerns and warranting further investigation of toxin production. These findings highlighted the need for targeted resistance strategies against the prevalent *Alternaria* species in the region.

## 1 Introduction

Mustard, scientifically known as *Brassica juncea* L. and commonly referred to as Indian mustard, is a prominent oilseed crop within the Brassica group. Despite its importance, mustard is severely affected by numerous fungal diseases, with *Alternaria* leaf blight (ALB) being one of the most destructive and widespread. ALB leads to significant losses in both yield (15–71%) and oil content (14–36%) (Bal and Kumar, 2014; Wang et al., 2021). In mustard, symptoms include dark brown to gray spots that may or may not be surrounded by a chlorotic halo (Siciliano et al., 2017). Concentric rings on the leaves reduce photosynthetic efficiency and ultimately result in abnormal seed development, thereby lowering both oil content and quality (Saharan et al., 2016). The genus Alternaria represents a diverse and ubiquitous group of fungi with a high degree of variability in spore shape and size, pathogenicity, and sporulation. Furthermore, conidial characteristics often overlap between closely related species, hindering the establishment of distinct and unambiguous species boundaries. Traditionally, species classification was based mainly on morphological characteristics, but this approach was influenced by environmental conditions (Simmons, 2007). Therefore, to overcome the limitations of morphological markers and complement morphological approaches to *Alternaria* taxonomy, various molecular markers have been used in systematic studies of *Alternaria species*. Recent molecular marker technologies have transformed genomic analyses of plant pathogens, facilitating their identification, early detection, and the study of genetic diversity. The internal transcribed spacer (ITS) region is the universal DNA barcode for fungal identification and has been widely used for species delimitation in the genus Alternaria (Kashyap et al., 2017; Zhang et al., 2017). However, because ITS alone often provides insufficient resolution for distinguishing closely related Alternaria species, additional protein-coding loci, including glyceraldehyde-3-phosphate dehydrogenase (GAPDH), RNA polymerase II second largest subunit (RPB2), translation elongation factor 1-α (TEF1), histone H3 (H3), and the Alternaria major allergen gene (Alt a1), have been increasingly employed to improve species identification and phylogenetic resolution (Woudenberg et al., 2015). Among these, the Alt a1 gene has been widely recognized as a useful marker for discriminating closely related Alternaria species owing to its higher sequence variability than the ITS region (Cramer and Lawrence, 2003; Hong et al., 2005). The polyketide synthase genes (*pksH* and *pksJ*) are involved in mycotoxin biosynthesis and contribute significantly to the pathogenicity and virulence of *Alternaria* species. Alternariol (AOH) and alternariol methyl ether (AME) are among the most common mycotoxins in *A*. *alternata* (Saha et al., 2012). ALB remains difficult to manage because durable and broad-spectrum host resistance is still unavailable in commercially cultivated Brassica varieties, despite the identification ofseveral resistant germplasm sources and resistance-associated loci (Singh et al., 2021; Mishra et al., 2024). The severity of Alternaria blight on oilseed brassicas varies from season to season, from region to region, and among individual crops in India (Chattopadhyay et al., 2005). Although ALB in brassicas is widespread and has a high economic impact under conducive weather conditions, there are no specific data on the number of causal species or detailed studies of the main aspects of the disease in mustard crops in the Bundelkhand region. Therefore, the present study aimed to investigate the variability and population diversity of Alternaria species occurring in the Bundelkhand region of Uttar Pradesh, India, with the goal of generating insights into the involvement of different Alternaria species in causing ALB in oilseed brassicas.

## 2 Materials and methods

### 2.1 Sampling and fungal strain isolation

**Figure 1:**
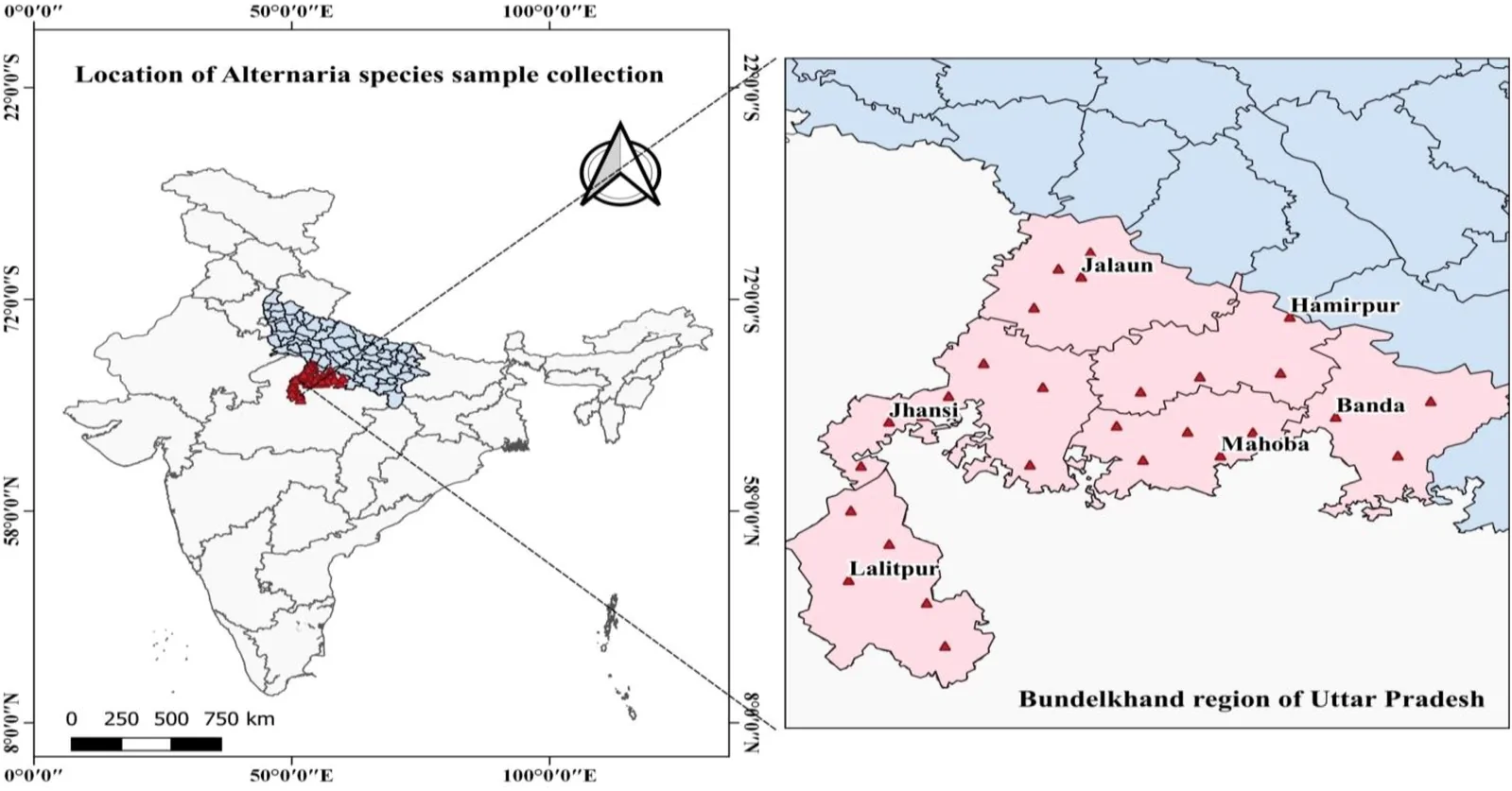
Geographical locations indicating sampling sites of Alternaria spp. Isolates.

During *the* 2023–2024 rabi season, a total of 28 mustard fields were selected and surveyed across 28 blocks within six administrative districts of the Bundelkhand region (**Figure 1**). From each field, 25 leaf samples infected with leaf blight were randomly collected across the entire field and brought to the laboratory for isolation. First, infected leaf tissues were cut into small pieces (2–3 mm) containing both infected and healthy portions. Excised leaf pieces were surface-sterilized using a 1% sodium hypochlorite solution for 1 min, rinsed three times in sterile distilled water, placed on potato dextrose agar (PDA) plates, and cultured at 25 ± 2°C for 5 days. The colonies growing on the leaf pieces were aseptically transferred to fresh PDA plates using a sterile needle for purification.

### 2.2 Cultural and morphological characterization of isolates

To investigate the morphological characteristics, 5-mm mycelial discs were cut from the 7-day-old cultures, placed centrally on fresh PDA plates, and incubated for 7 days at 25°C. Cultural characteristics such as colony color, margin, and general appearance were recorded. After 12 days, conidial length and breadth and the number of septa per conidium were measured under a light microscope (Olympus BX51, Olympus Corporation, Tokyo, Japan), and all images were captured at ×40 magnification.

### 2.3 Pathogenicity test

The virulence of 28 purified isolates was tested on detached healthy leaves of the susceptible cultivar ‘Rohini’ using the disc inoculation method under laboratory conditions. The leaves were washed under running tap water, rinsed with autoclaved water, and wiped with 70% ethanol. A 5-mm agar disc from a 7-day-old culture was cut and aseptically placed on the leaf surface under laminar airflow. The inoculated leaves were placed on blotting paper in sterilized Petri dishes, with one leaf per dish, and maintained in a biochemical oxygen demand (BOD) incubator at 25 ± 2°C. The blotting paper was kept moist by spraying it with autoclaved distilled water. Observations on the onset of disease symptoms were recorded at 24-h intervals for up to 120 h after pathogen inoculation. An uninoculated control was maintained separately for each isolate. The pathogens were reisolated from the inoculated leaves to prove Koch’s postulates.

### 2.4 Isolation of genomic DNA, PCR amplification, and sequencing

Based on the pathogenicity results, the 18 highly virulent isolates representing the three identified *Alternaria* species were selected for subsequent molecular characterization, phylogenetic analysis, and haplotype network analysis. Total genomic DNA was extracted from the 18 highly virulent isolates using the cetyltrimethylammonium bromide (CTAB) technique (Li et al., 2013). The isolates were cultured in 50 mL of potato dextrose broth in a BOD incubator shaker at 100 rpm and 25 ± 2°C for 5–6 days. The mycelial balls were harvested using filtration and dried for DNA extraction. The concentration and purity of DNA were assessed using a NanoDrop instrument (Thermo Fisher™ Scientific, Mumbai, Maharashtra, India). The quality of the genomic DNA was tested using 0.8% agarose gel electrophoresis (iGeneLabserve™, New Delhi, India), and the DNA was stored at −20°C until further use. Two partial regions of the isolated fungal genome were amplified: the ITS region using the ITS-1 and ITS-4 primer pair and the *Alt a1* region (*Alt a1 allergen* gene) using the Alt-forward and Alt-reverse primer pair, following the amplification conditions in **Table 1**. PCR amplification was performed in 25-μL reaction mixtures containing 1 μL of DNA template, 12.5 μL of Mastermix (HiMedia®, Mumbai, Maharashtra, India), 1 μL of each primer, and 9.5 μL of double-distilled water (ddH_2_O) in a Thermocycler (Applied Biosystems™, New Delhi, India). All PCR products were sequenced using the Sanger method at Eurofins Genomics, Bangalore, India.

**Table 1:** Primers and PCR conditions used.

Primer	Primer DNA sequence	PCR conditions	Amplicon size	Reference
ITS1	5’ TCC GTA GGT GAA CCTGCG G 3′	94°C for 5 min; 94°C for 30 s, 48°C for 30 s, and 72°C for 90 s for 35 cycles, with a final extension at 72°C for 7 min	550 bp	Woudenberg et al. (2013)
ITS4	5’ GCT GCG TTC TTC ATCGAT GC 3′			
AltF	5’ATG CAG TTC ACC ACC ATC GC 3′	95°C for 5 min; 95°C for 30 s, 58°C for 30 s, and 72°C for 30 s for 35 cycles, with a final extension at 72°C for 7 min	510 bp	Hong et al. (2005)
AltR	5’ACG AGG GTG AYG TAG GCG TC 3′			
PksH	5’GTCAACCCTCTCACACCAAC3′	94°C for 5 min; 94°C for 30 s, 62°C for 30 s, and 72°C for 30 s for 35 cycles, with a final extension at 72°C for 7 min	290 bp	Saleem and El-Shahir (2022)
PksH	5’GACGCATCGCTTCAATAGCC3′			
PksJ	5’GTCCCAAATTCCTACCCTCAC3′	94°C for 5 min; 94°C for 30 s, 62°C for 30 s, and 72°C for 30 s for 35 cycles, with a final extension at 72°C for 7 min	260 bp	Saleem and El-Shahir (2022)
PksJ	5’GATAGCCATCGAAAGCATTCCC3′			

### 2.5 Analysis of phylogenetic relationships and haplotype network

The obtained sequence data were subjected to BLASTn analysis using the National Center for Biotechnology Information (NCBI) website (https://blast.ncbi.nlm.nih.gov/Blast.cgi) following alignment using ClustalW in Molecular Evolutionary Genetics Analysis (MEGA) version 11.0. Sequences of closely related *Alternaria* strains were retrieved from GenBank to construct datasets for phylogenetic analysis. The GenBank sequences were combined with the sequences obtained in this study and concatenated, and a phylogenetic tree was constructed using the maximum likelihood (ML) algorithm based on the Tamura–Nei model in MEGA version 11.0 (Tamura et al., 2021), using the bootstrap method with 1,000 repetitions. Similarly, the nucleotide sequences of the ITS and Alt a1 genes were concatenated for haplotype network analysis of the *Alternaria* isolates using PopART version 1.7 (Leigh et al., 2015) and the Templeton–Crandall–Sing (TCS) method. Sequences were aligned and trimmed to the same length, and ambiguous regions were excluded before analysis. In the network, haplotypes were represented as nodes, with node size proportional to haplotype frequency, while different colors were used to differentiate among *Alternaria* species.

### 2.6 Detection of *pksH* and *pksJ* genes associated with toxin biosynthesis

Based on the species identification and pathogenicity results, only the virulent A. alternata isolates were selected for pksH and pksJ screening to assess their genetic potential for toxin biosynthesis. The *pksH* and *pksJ* genes, which are associated with the biosynthesis of Alternaria toxins, were amplified from the genomic DNA of the selected *A. alternata* isolates using gene-specific primer pairs in a 25 µL PCR reaction under the amplification conditions presented in **Table 1**. The PCR products were separated on a 1% agarose gel in 0.5 × Tris-acetate-EDTA (TAE) buffer, visualized after ethidium bromide staining under UV illumination, and sequenced using the Sanger method. High-quality consensus sequences were assembled in BioEdit software and compared with NCBI GenBank entries using BLASTn. Phylogenetic relationships were inferred using MEGA version 11.0 and the ML method with 1,000 bootstrap repetitions, including reference sequences of *A. alternata* retrieved from GenBank.

## 3 Results

### 3.1 Survey and collection of infected leaf samples

A comprehensive survey was conducted to assess the prevalence and spatial distribution of *Alternaria* blight in mustard during the 2023–2024 and 2024–2025 rabi seasons. Diseased leaf samples exhibiting typical *Alternaria* blight symptoms were randomly collected. Twenty-eight isolates were collected from different blocks across six districts in the Bundelkhand region, namely, Jhansi, Lalitpur, Banda, Mahoba, Jalaun, and Hamirpur (**Figure 1**; **Table 2**).

**Table 2:** Collection of infected leaf samples from different districts of the Bundelkhand region, Uttar Pradesh.

District	Block	Isolate code	Latitude	Longitude	Date of collection	Host (plant part)
Jhansi	Moth	Alt.1	25.722812	78.944648	24/12/2023	*B. juncea* (Leaf)
Jhansi	Gursarai	Alt.2	25.579756	79.171877	24/12/2023	*B. juncea* (Leaf)
Jhansi	Bamor	Alt.3	25.663403	79.048949	26/12/2023	*B. juncea* (Leaf)
Jhansi	Badagoan	Alt.4	25.522326	78.436086	26/12/2023	*B. juncea* (Leaf)
Jhansi	Babina	Alt.5	25.485539	78.427297	28/12/2023	*B. juncea* (Leaf)
Jhansi	Chirgoan	Alt.6	25.558965	78.804284	28/12/2023	*B. juncea* (Leaf)
Jhansi	Jhansi	Alt.7	25.516854	78.548648	15/01/2024	*B. juncea* (Leaf)
Lalitpur	Talbehat	Alt.8	25.005046	78.447554	16/01/2024	*B. juncea* (Leaf)
Lalitpur	Jakhaura	Alt.9	24.829845	78.463291	16/01/2024	*B. juncea* (Leaf)
Lalitpur	Bar	Alt.10	24.783581	78.537326	17/01/2024	*B. juncea* (Leaf)
Lalitpur	Mehroni	Alt.11	24.558954	78.679568	17/01/2024	*B. juncea* (Leaf)
Lalitpur	Madawara	Alt.12	24.440207	78.659768	18/01/2024	*B. juncea* (Leaf)
Lalitpur	Birdha	Alt.13	24.730332	78.524278	18/01/2024	*B. juncea* (Leaf)
Banda	Barokhar	Alt.14	25.436386	80.37438	24/01/2024	*B. juncea* (Leaf)
Banda	Mahua	Alt.15	25.404935	80.417220	24/01/2024	*B. juncea* (Leaf)
Banda	Tindwari	Alt.16	25.576980	80.477270	28/01/2024	*B. juncea* (Leaf)
Banda	Bisanda	Alt.17	25.484555	80.675027	28/01/2024	*B. juncea* (Leaf)
Banda	Baberu	Alt.18	25.591355	80.607131	28/01/2024	*B. juncea* (Leaf)
Mahoba	Kabrai	Alt.19	25.330406	79.830374	04/02/2024	*B. juncea* (Leaf)
Mahoba	Charkhari	Alt.20	25.354225	79.749477	04/02/2024	*B. juncea* (Leaf)
Mahoba	Jaitpur	Alt.21	25.339521	79.605815	05/02/2024	*B. juncea* (Leaf)
Mahoba	Panwari	Alt.22	25.408815	79.505011	05/02/2024	*B. juncea* (Leaf)
Jalaun	Konch	Alt.23	26.019779	79.148735	20/01/2025	*B. juncea* (Leaf)
Jalaun	Madhogarh	Alt.24	26.212885	79.169012	21/01/2025	*B. juncea* (Leaf)
Jalaun	Jalaun	Alt.25	26.154468	79.27396	21/01/2025	*B. juncea* (Leaf)
Hamirpur	Rath	Alt.26	25.491633	79.501848	12/02/2025	*B. juncea* (Leaf)
Hamirpur	Muskara	Alt.27	25.641389	79.776496	12/02/2025	*B. juncea* (Leaf)
Hamirpur	Maudaha	Alt.28	25.699492	80.140885	13/02/2025	*B. juncea* (Leaf)

### 3.2 Morphological characterization

**Figure 2:**
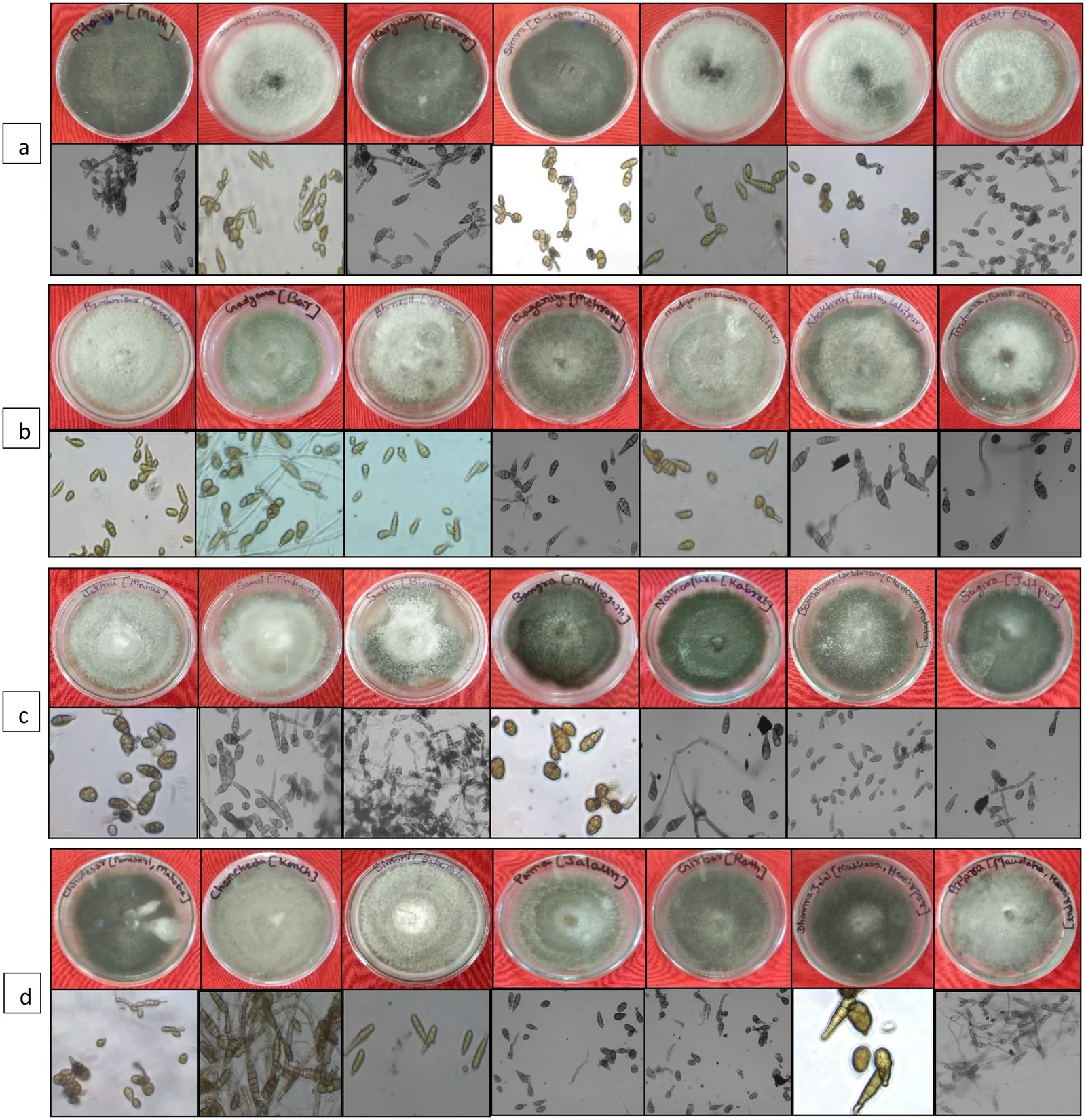
Colony morphology of *Alternaria* spp. on PDA (15 days old culture) and conidia formation on V8 agar medium. **(a)** Alt1-7, **(b)** Alt 8-14, **(c)** Alt15-21, **(d)** Alt 22-28.

**Figure 3:**
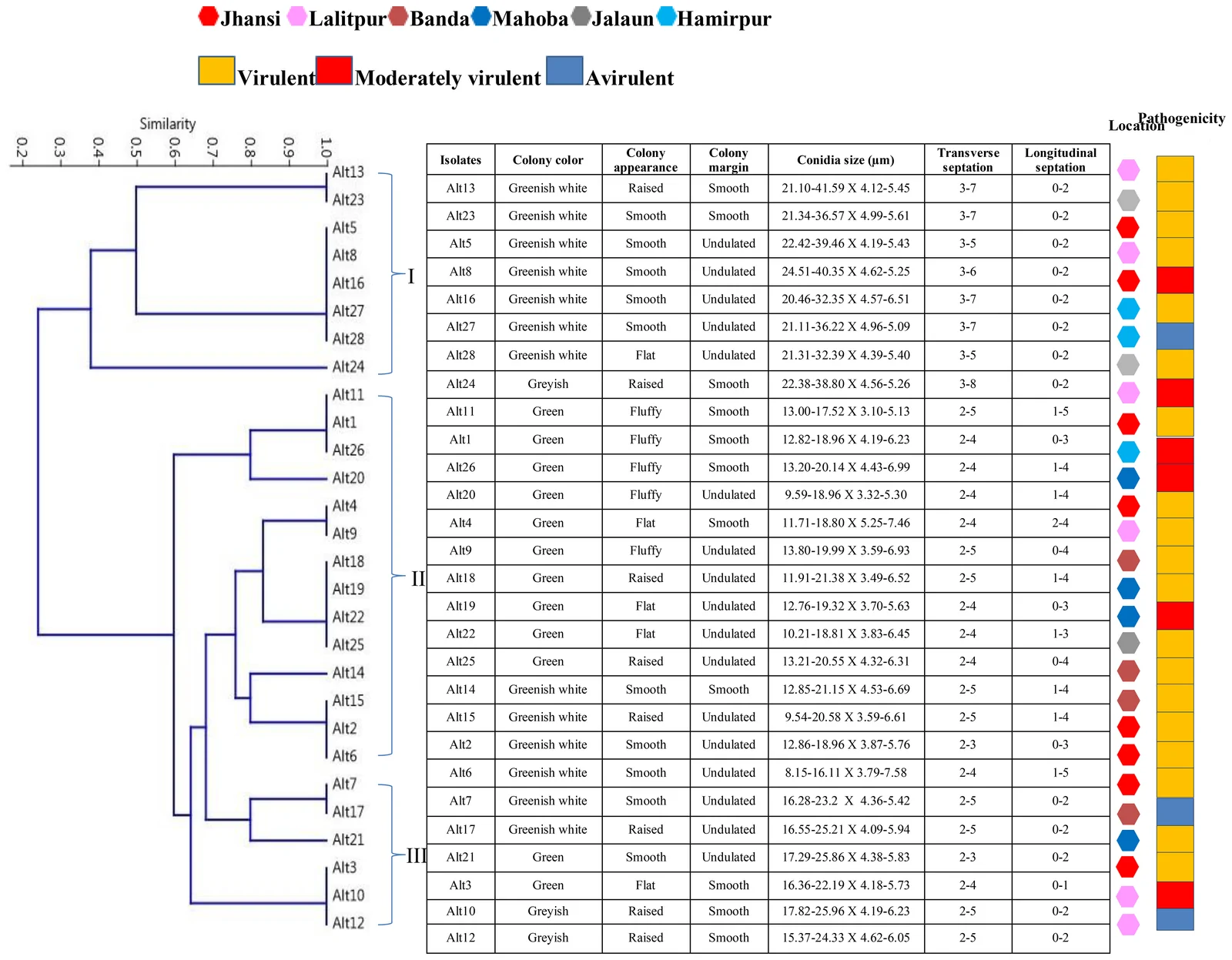
Dendrogram derived from morphological characteristics of 28 isolates of *Alternaria* spp. grown on PDA medium, based on colony color, appearance, margin, conidial size (µm), and the numbers of transverse and longitudinal septa.

**Figure 4:**
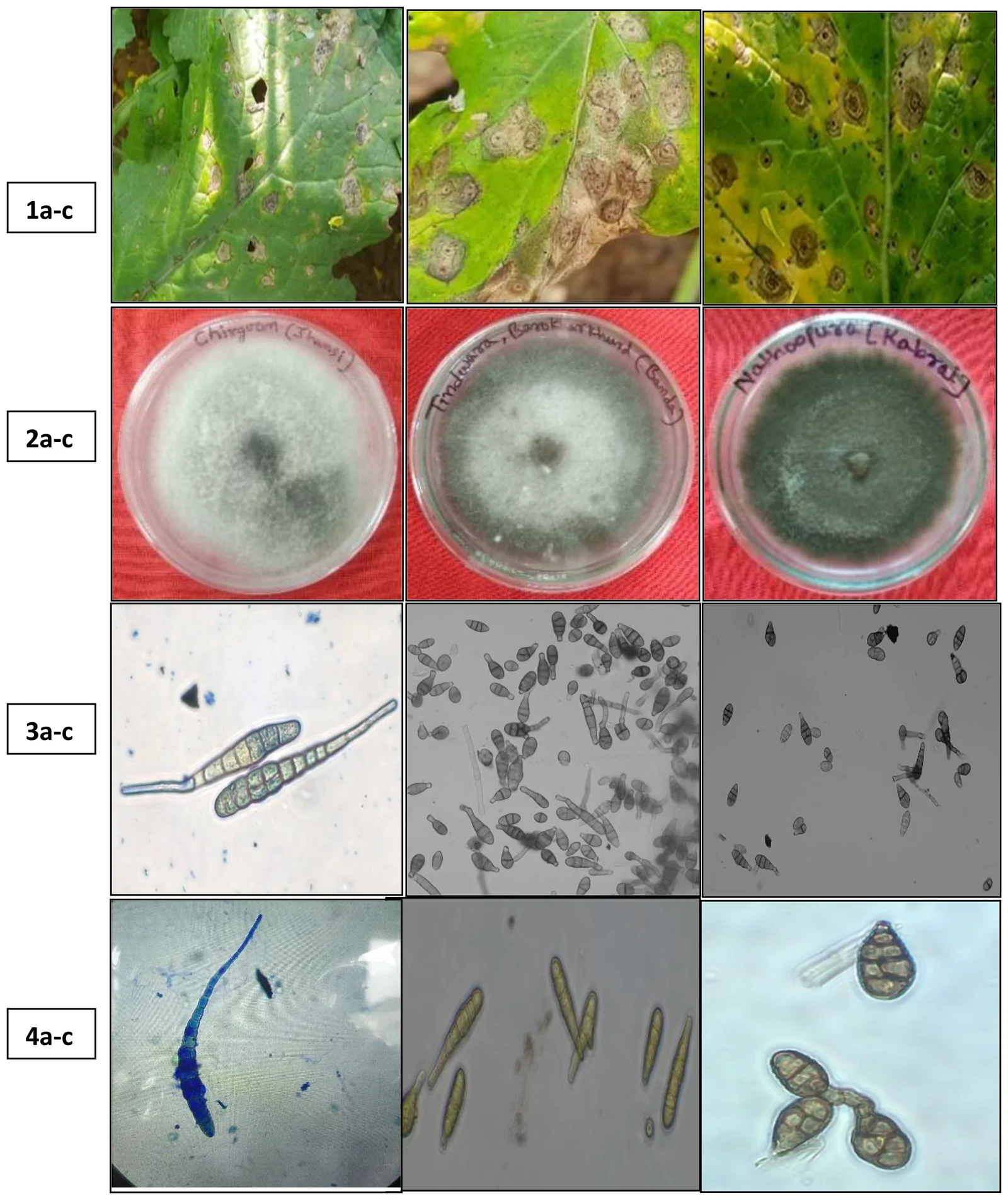
**(1a-c)** Symptoms of leaf spots on mustard; **(2a-c)** 7-day-old culture on PDA; **(3a-c, 4a-c)** conidia; a –*A. brassicae*, b - *A*. *brassicicola*, c - *A*. *alternata*.

Alternaria isolates showed variability in numerous phenotypic characteristics, such as colony appearance, color, margin, conidial size (µm), and longitudinal and transverse septation of conidia. The isolates displayed pronounced differences in colony color, including green, greenish white, and grayish. Mycelial appearance varied considerably, ranging from fluffy and raised to flat and smooth, and colony margins were either smooth or undulated. Microscopic observations of conidial morphology at ×40 magnification revealed variability in conidial size and the number of septa. A significant variation in their color and conidia among different isolates is depicted in **Figure 2**. Isolates were divided into distinct clusters based on colony appearance, color, margin, conidial size, and longitudinal and transverse septation of conidia. Based on morphological features, all 28 isolates were grouped into three clusters that were identified as species (**Figure 3**). Cluster I consisted of eight isolates with greenish white to grayish colony color, colony appearance ranging from smooth to raised, and mycelial margins ranging from smooth to undulated on PDA. Conidial size ranged from 21.10–41.59 × 4.12–6.51 μm, and the numbers of transverse and longitudinal septa ranged from 3–9 and 0–2, respectively. Cluster II comprised 14 isolates with green to greenish white colonies. Colony appearance was highly variable, ranging from fluffy and raised to flat and smooth, and colony margins ranged from smooth to undulated. Conidial size ranged from 8.15–21.38 × 3.10–6.61 μm, and the numbers of transverse and longitudinal septa ranged from 1–6 and 1–4, respectively. Six isolates in Cluster III exhibited green to greenish white or grayish colonies, raised, smooth, or flat colony appearances, and smooth to undulated margins. Conidial size ranged from 15.37–25.86 × 4.09–6.23 μm, and the numbers of transverse and longitudinal septa ranged from 1–6 and 0–2, respectively. The first group of 8 large-spored isolates was identified as *A. brassicae*; the second group, morphologically the most variable one, consisted of 14 isolates of *A. alternata;* and the third group of 6 small-spored isolates was *A. brassicicola*, according to Simmons (2007) (**Table 3**; **Figures 3**, **4**).

**Table 3:** Description of morphological characteristics of *Alternaria* spp. from mustard.

	Species (number of isolates)		
Morphological characteristics	*A*. *brassicae* (8)	*A*. *alternata* (14)	*A*. *brassicicola* (6)
Mycelial texture and shape	Cottony, circular	Cottony, circular	Cottony, circular
Colony color	Greenish white	Green	Grayish green
Colony margin	Undulated	Smooth or undulated	Smooth or undulated
Conidial shape	Mostly obclavate	Obclavate to long ellipsoid	Ellipsoid or obclavate
Conidial length (μm)	21–45	8.0–20	18–26
Conidial breadth (μm)	4.12–5.64	5.22–7.62	4.22–5.82
Number of transverse septa (min.–max.)	3–9	1–6	1–6
Number of longitudinal septa	0–2	1–4	0–2

### 3.3 *In vitro* pathogenicity test of *Alternaria* isolates

**Figure 5:**
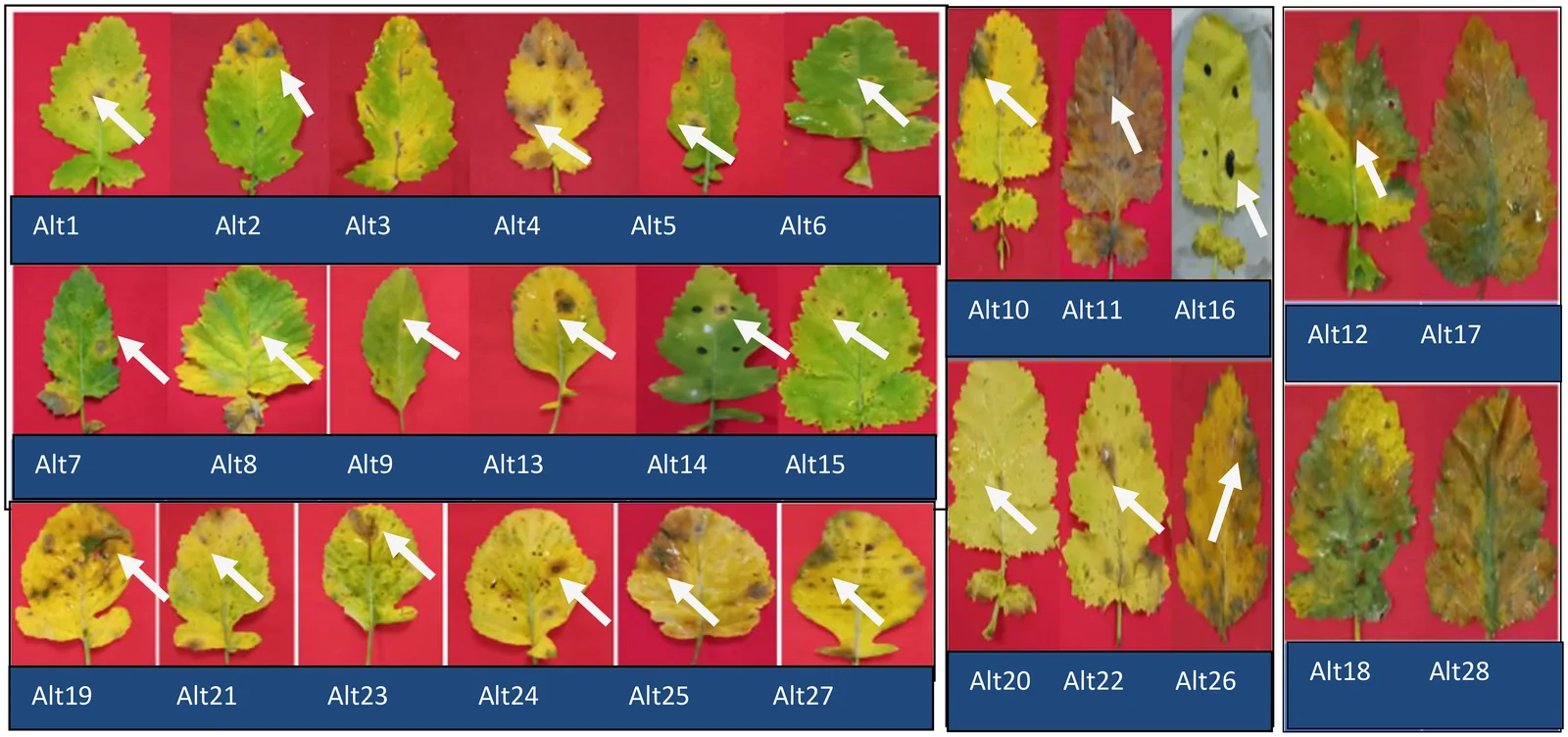
Pathogenicity test showing symptoms caused by highly virulent isolates (Alt1, 2, 3, 4, 5, 6, 7, 8, 9, 13, 14, 15, 19, 21, 23, 24, 25, and 27), moderately virulent isolates (Alt10, 11, 16, 20, 22, and 26), and less virulent isolates (Alt12, 17, 18, and 28).

All 28 *Alternaria* isolates were evaluated for pathogenicity based on lesion size on the mustard cultivar ‘Rohini’ during *the* 2023–2024 rabi season. Of the 28 isolates, 18 isolates (Alt1, 2, 3, 4, 5, 6, 7, 8, 9, 13, 14, 15, 19, 21, 23, 24, 25, and 27) were highly virulent and produced the largest lesions, with an average diameter of 10.0–15.0 mm, 5 days after inoculation; 6 isolates (Alt 10, 11, 16, 20, 22, and 26) caused yellowing symptoms on the inoculated leaves and were categorized as moderately virulent; and the remaining 4 isolates (Alt 12, 17, 18, and 28) did not produce any symptoms and were classified as less virulent (**Figure 5**).

### 3.4 Molecular identification of *Alternaria* isolates

**Figure 6:**
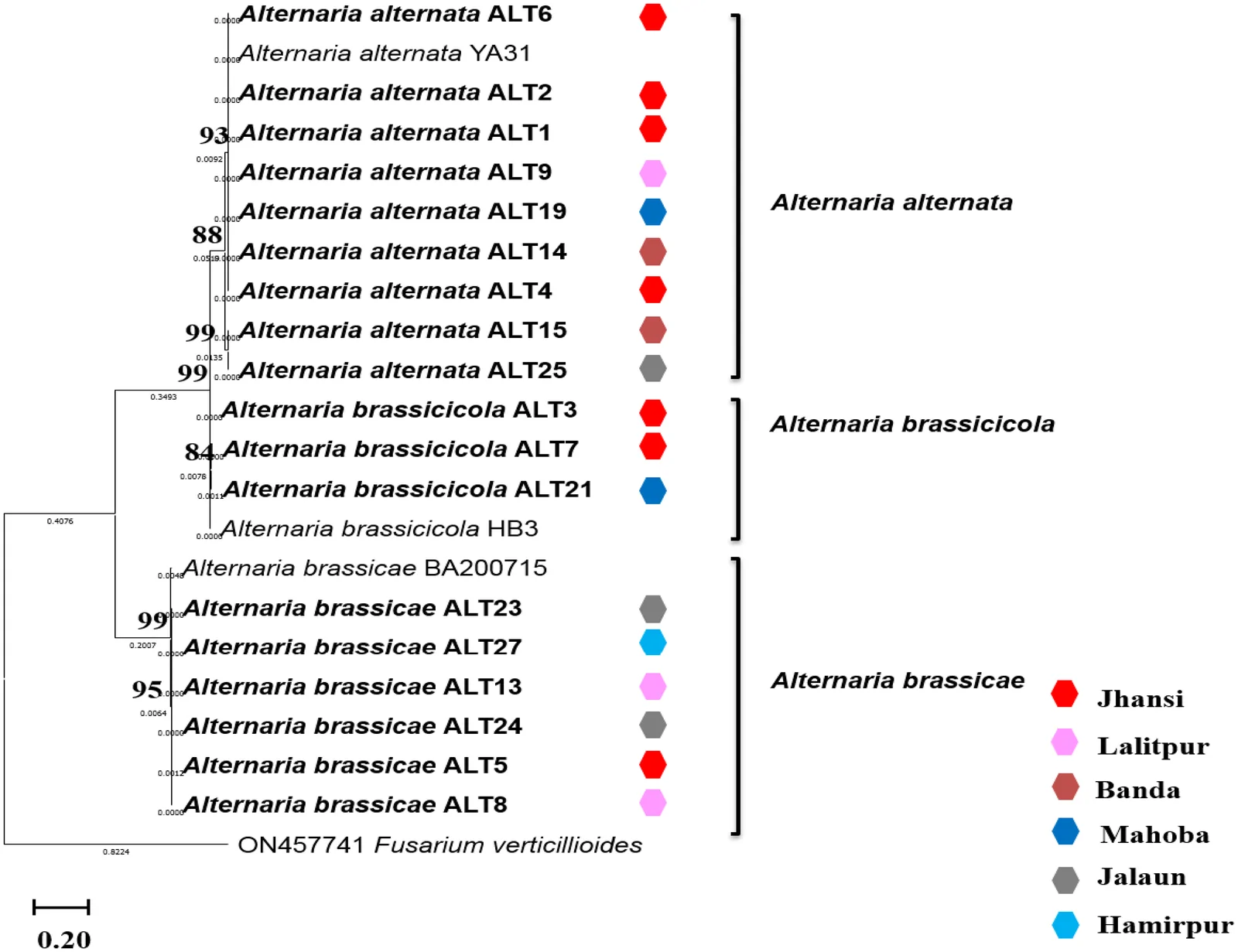
Phylogenetic relationship derived fromconcatenated(*ITS* + *Alt a1*) gene sequences of *Alternaria* spp. virulent strains by MEGA XI software using ML method and the best-fit model (TN93+G+I) based on 1,000 bootstrap replicates. The tree was rooted using sequences of *Fusarium verticillioides*. Support values (greater than 50%) are displayed.

In PCR analysis, the fragments of genes, ITS (~550 bp) and *Alt a1* (~510 bp) were amplified in all 18 most virulent isolates. All sequences were submitted to the NCBI GenBank database (the corresponding accession numbers are provided in **Table 4**) and searched using BLASTn for closely related sequences. Phylogenetic and molecular evolutionary analyses were performed for the collected *Alternaria* isolates and previously reported *A*. *brassicae*, *A*. *brassicicola*, and *A*. *alternata* isolates using the maximum likelihood (ML) method, while *Fusarium verticillioides* was used as an outgroup to validate the tree (**Figure 6**). The phylogenetic tree based on concatenated ITS and *Alt a1* sequences demonstrated that the virulent strains recovered from different locations in the Bundelkhand region clustered together, confirming the homology-based identification through the formation of three distinct clades with moderate-to-high bootstrap values (**Figure 6**). The *A*. *alternata* isolates, namely, Alt 1, 2, 4, 6, 9, 14, 15, 19, and 25, were closely related to and grouped with the *A*. *alternata* reference strain MZ330635 YA31; the *A*. *brassicicola* isolates, namely, Alt 3, 7, and 21, were closely related to and grouped with the *A*. *brassicicola* reference strain OP096435 HB3; and the *A*. *brassicae* isolates, namely, Alt 5, 8, 13, 23, 24, and 27, were closely related to and grouped with the *A*. *brassicae* reference strain OK064153 BA200715.

**Table 4:** Selected isolates used in this study and their corresponding GenBank accession numbers used in phylogenetic analyses.

Species	District	Block	Isolates	GenBank accession number			
				*ITS*	*Alta1*	*PksH*	*PksJ*
*A. brassicae*	Jhansi	Babina	Alt.5	PQ231108	PV258717	–	–
*A. brassicae*	Lalitpur	Talbehat	Alt.8	PQ197803	PV335432	–	–
*A. brassicae*	Lalitpur	Birdha	Alt.13	PQ549906	PV334935	–	–
*A. brassicae*	Jalaun	Konch	Alt.23	PQ482359	PV334936	–	–
*A. brassicae*	Jalaun	Madhogarh	Alt.24	PQ468465	PV296230	–	–
*A. brassicae*	Hamirpur	Muskara	Alt.27	PQ345721	PV081784	–	–
*A. brassicicola*	Jhansi	Bamor	Alt.3	PQ222681	PV296229	–	–
*A. brassicicola*	Jhansi	Jhansi	Alt.7	PQ510337	PV334934	–	–
*A. brassicicola*	Mahoba	Jaitpur	Alt.21	PQ516740	PV067054	–	–
*A. alternata*	Jhansi	Moth	Alt.1	PQ197634	PV081785	PV786833	PV798443
*A. alternata*	Jhansi	Gursarai	Alt.2	PQ556156	PV057425	PV779693	PV077734
*A. alternata*	Jhansi	Badagoan	Alt.4	PQ198413	PV258716	PV786835	PV798444
*A. alternata*	Jhansi	Chirgoan	Alt.6	PQ345477	PV057426	PV167494	PV693339
*A. alternata*	Lalitpur	Jakhaura	Alt.9	PQ345719	PV067051	PV172432	PV164363
*A. alternata*	Banda	Barokhar	Alt.14	PQ197633	PV258718	PV786834	PV798445
*A. alternata*	Banda	Mahua	Alt.15	PQ345720	PV067053	PV167495	PV167491
*A. alternata*	Mahoba	Kabrai	Alt.19	PQ222683	PV067052	PV169307	PV167493
*A. alternata*	Jalaun	Jalaun	Alt.25	PQ345478	PV335431	PV167522	PV16749

### 3.5 Haplotype analyses

**Figure 7:**
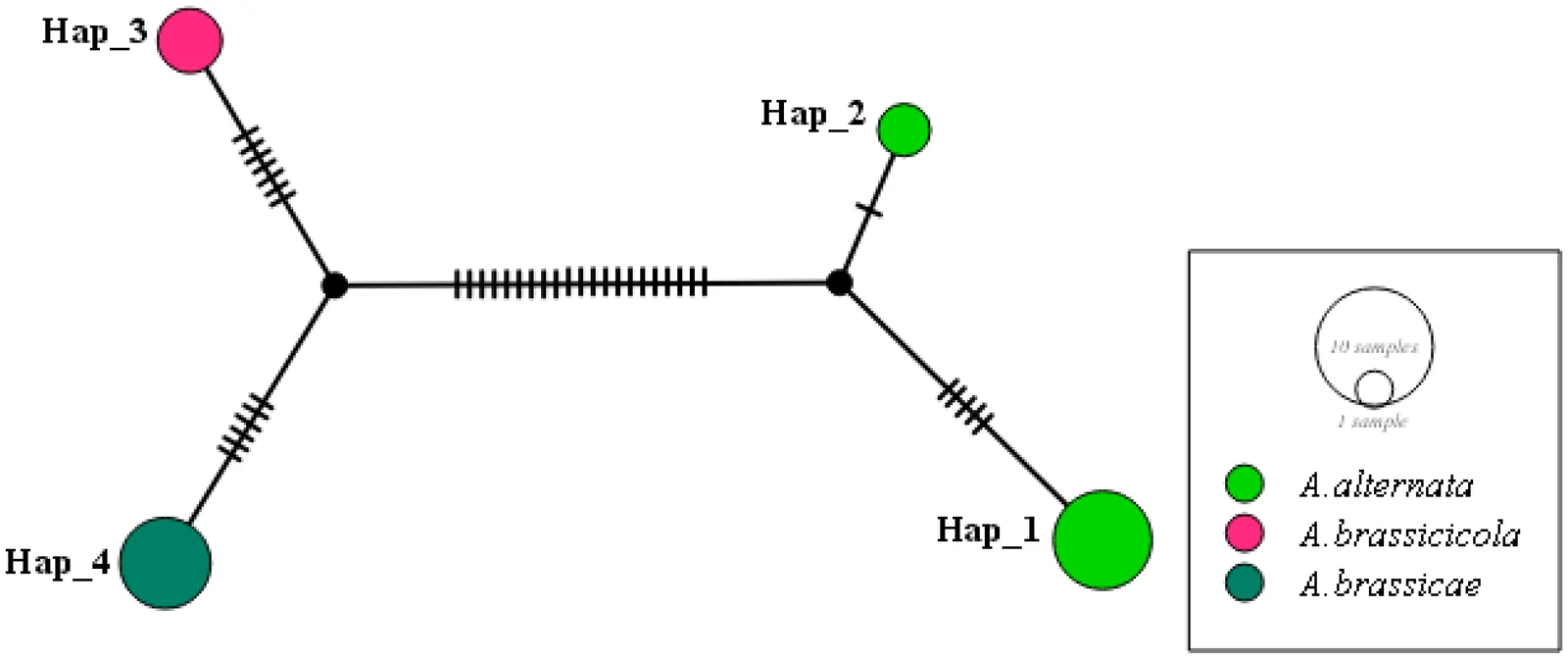
Haplotypes network based on TCS networkderived from concatenated sequence of *ITS* and *Alta1* genes of *Alternaria* isolates obtained from Bundelkhand region (U.P), India. Each haplotype is represented with a circle and their size is proportional to the haplotype frequency.

To determine the evolutionary relationships among haplotypes, a TCS network using the most parsimonious connections was constructed based on concatenated *ITS* and *Alt a1 gene sequences* (**Figure 7**). The results of the network were similar to the taxonomic grouping derived from the phylogenetic analysis. We identified four haplotypes (Hap_1–Hap_4) among the analyzed isolates, which formed different groups corresponding to *A*. *alternata*, *A*. *brassicae*, and *A*. *brassicicola*. The identified haplotypes clearly separated these species into independent clusters, indicating significant genetic divergence. Hap_1, which comprised *A*. *alternata* isolates, was the most frequent and centrally distributed haplotype, indicating its dominance within the population. In contrast, the smaller haplotypes (Hap_2, Hap_3, and Hap_4), which were mainly associated with *A. alternata, A. brassicicola, and A. brassicae*, respectively, formed distinct but closely related nodes, suggesting low-to-moderate genetic variation among these groups. The overall network showed clear genetic differentiation among species, with haplotypes separated by a small number of mutational steps and no overlap between species clusters. This clear separation suggested restricted gene flow and supported independent evolutionary lineages among the three *Alternaria* species. It also provided a simplified and clear visualization of genetic relationships, supported the results of the phylogenetic analysis, and confirmed species-level divergence.

### 3.6 Detection of *pksH* and *pksJ* genes in *Alternaria alternata*

**Figure 8:**
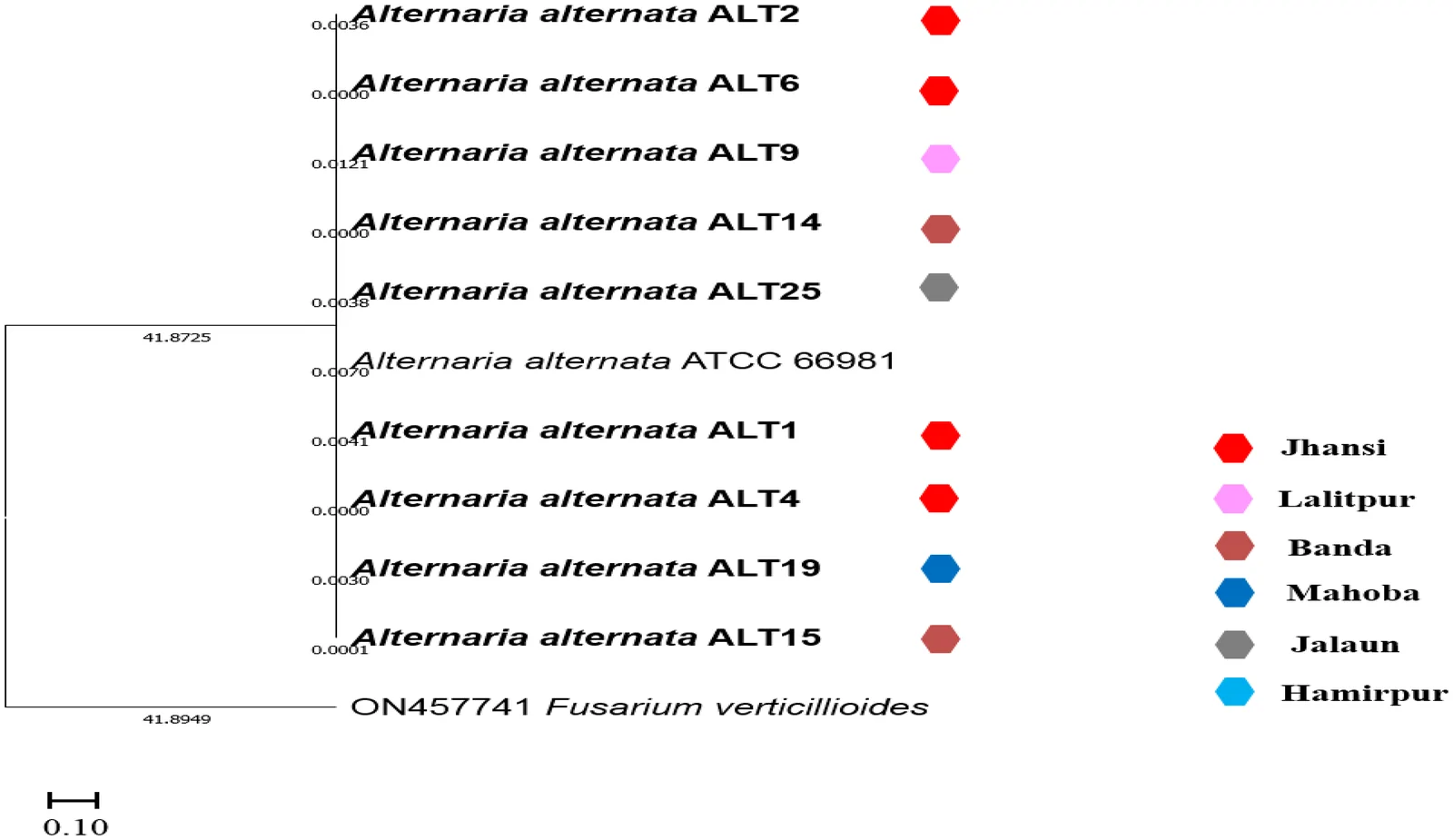
Phylogenetic relationship among nine *A.alternata* isolates based on concatenated (PksJ + PksH) sequences by ML method based on 1,000 bootstrap replicatesusing MEGA 11.0 in which associated taxa clustered together.

Polyketide synthase genes (*pksH* and *pksJ*) were successfully amplified from the genomic DNA of all *A*. *alternata* isolates, producing clear and reproducible bands of the expected sizes (~290 bp for *pksH* and ~260 bp for *pksJ*), confirming the presence of genes associated with toxin biosynthesis. Sequencing of purified amplicons yielded high-quality reads and BLASTn analysis showed 100% homology with reference *A*. *alternata* sequences available in GenBank, validating gene-specific amplification. Phylogenetic analysis based on concatenated *pksH* and *pksJ* sequences using the ML method with 1,000 bootstrap replicates revealed that the isolates clustered closely with previously reported mycotoxin-producing *A*. *alternata* strains (reference species: JX103645 ATCC strain), with strong support (bootstrap = 100%) for the grouping of the test isolates, indicating high genetic relatedness (**Figure 8**). The detection of these genes suggests the genetic potential for toxin biosynthesis; however, the production of Alternaria toxins was not directly assessed in this study.

## 4 Discussion

The present study describes the prevalence and distribution of *Alternaria* species causing leaf blight in mustard in the Bundelkhand region, Uttar Pradesh. Morphological characterization revealed considerable variability among isolates in colony color, texture, margin, conidial dimensions, and septation patterns. Based on these features, isolates were classified into three groups corresponding to *A*. *brassicae*, *A*. *brassicicola*, and *A*. *alternata*. Such morphological variability has been widely reported and is often influenced by environmental conditions and host interactions (Simmons, 2007; Saharan et al., 2016). Notably, *A*. *brassicae* was identified as a large-spored species, *A*. *brassicicola* as a small-spored one, while *A*. *alternata* displayed a high degree of variability. Molecular analyses are essential for revealing complex species composition and inter- and intraspecies genetic relationships. While morphological and cultural characteristics are often unreliable for identification because of their variability (Lawrence et al., 2016), genetic analyses supported the results of morphological identification and enabled discrimination among the groups. The pathogenicity test revealed that 18 isolates were highly virulent and produced lesions on leaves of the susceptible cultivar ‘Rohini’, whereas 6 isolates were moderately virulent and 4 isolates were less virulent. Based on molecular characterization, the 18 highly virulent isolates comprised six *A*. *brassicae*, three *A*. *brassicicola*, and nine *A*. *alternata*. The predominance of highly virulent isolates among these three species is consistent with previous reports demonstrating that multiple Alternaria species associated with oilseed brassicas are capable of causing severe disease symptoms under favorable conditions (Meena et al., 2017).

In this study, molecular analyses using concatenated ITS and *Alt a1 sequences* provided robust species delimitation and validated the morphological identification. The maximum likelihood tree formed three well-supported clades corresponding to *A*. *brassicae*, *A*. *brassicicola*, and *A*. *alternata*, consistent with multilocus phylogenetic analyses reported in previous taxonomic studies (Hong et al., 2005; Somma et al., 2019). Similarly, Aneja et al. (2014) reported the presence of *A*. *alternata*, *A*. *brassicae, and A*. *brassicicola* on oilseed brassicas, with *A*. *brassicae* as the predominant species. The clustering of Bundelkhand isolates with reference strains reported globally highlights the genetic relatedness and potential genetic stability of these pathogens across geographical regions. This study is the first to report the involvement of multiple *Alternaria* species in causing ALB in mustard.

The haplotype network analysis of *Alternaria* species in the present study revealed the genetic structure and evolutionary relationships within and among species. A total of four haplotypes were identified, forming distinct clusters corresponding to *A*. *brassicae*, *A*. *brassicicola*, and *A*. *alternata* and indicating clear species-level differentiation, which was also supported by the phylogenetic analysis. Further network analysis revealed a low level of intraspecific variation in *A*. *brassicicola*, with most isolates grouped within a single haplotype or closely related haplotypes. This pattern reflects a high level of genetic homogeneity and is consistent with previous reports in which only a single *ITS* haplotype of *A*. *brassicicola* has been reported across diverse geographical regions and hosts, including rapeseed-mustard in Serbia, canola in Canada, and cruciferous crops in India (Saharan et al., 2016; Akram et al., 2019; Blagojević et al., 2020). This variability may be attributed to the conserved nature of the genomic regions analyzed or limited evolutionary divergence within this species.

Similarly, *A*. *brassicae* isolates in the present study exhibited moderate-to-low haplotype diversity, supporting previous reports of very low or poorly characterized genetic variation in *ITS* and protein-coding regions such as GAPDH (Saharan et al., 2016). This limited genetic variability suggests a relatively stable population structure and may indicate a narrower adaptive range compared with other *Alternaria* species. On the other hand, *A*. *alternata* showed comparatively higher haplotype diversity, which may be due to its wide host range, ecological adaptability, and genetic plasticity, as reported in previous studies. Overall, the haplotype network demonstrated clear but not absolute genetic separation among species, with haplotypes connected by a limited number of mutational steps. This indicated restricted gene flow alongside partial genetic relatedness, and the network structure was largely consistent with the phylogenetic analysis, supporting species delimitation while also highlighting subtle genetic connectivity among *Alternaria* species.

Although multiple Alternaria species were recovered from the surveyed mustard fields, their ecological interactions during disease development remain unclear. Whether these species interact synergistically, competitively, or independently under natural infection conditions was beyond the scope of the present study. Future investigations involving controlled single- and mixed-species inoculation experiments would help determine their individual and combined contributions to disease severity and epidemic development.

Alternaria species produce more than 60 secondary metabolites. Alternariol (AOH) and alternariol monomethyl ether (AME) are among the most common mycotoxins in *A*. *alternata*. Polyketide synthases (PKSs) are important enzymes involved in the biosynthesis of these toxins. The pksH and *pksJ* genes are among the essential genes associated with toxin production (Saha et al., 2012). A major outcome of this study is the detection of polyketide synthase genes (*pksH* and *pksJ*) in all *A*. *alternata* isolates. The consistent amplification of pksH and pksJ genes, together with their phylogenetic clustering with known toxigenic strains, suggests the genetic potential of these isolates to synthesize Alternaria toxins such as alternariol (AOH) and alternariol monomethyl ether (AME). However, the expression of these genes and the production of AOH and AME in infected mustard tissues were not investigated in the present study. Previous studies have demonstrated that PKS genes play a crucial role in virulence, host colonization, and secondary metabolite production in *A*. *alternata* (Saha et al., 2012; Estiarte et al., 2016; Wenderoth et al., 2019). The presence of both genes in all *A*. *alternata* isolates suggests a conserved genetic potential for toxin biosynthesis, which may contribute to disease severity and pose potential risks to oilseed crop safety, echoing similar concerns in other Brassicaceae crops such as cabbage, cauliflower, broccoli, and radish (Saha, 2012; Saleem and El-Shahir, 2022). The identification of *A*. *alternata* isolates with the genetic potential for toxin biosynthesis on mustard in this region represents a significant finding, indicating an emerging threat that warrants further ecological and epidemiological investigation. Future studies integrating gene expression analyses with quantitative toxin profiling will be essential to determine whether pksH and pksJ are actively expressed during infection and to establish the contribution of alternariol (AOH) and alternariol monomethyl ether (AME) to virulence in the mustard-Alternaria pathosystem.

Although the present study provides comprehensive insights into the diversity, pathogenicity, and toxigenic potential of the Alternaria species complex associated with ALB of mustard, the cellular and molecular mechanisms underlying host responses to these pathogens remain largely unexplored. Emerging approaches such as single-cell and spatial transcriptomics, together with studies on phytohormone-mediated defense signaling, may provide valuable insights into host–pathogen interactions and the molecular basis of host susceptibility and resistance, thereby facilitating the identification of candidate molecular targets for resistance breeding (Ali et al., 2026, 2025).

## 5 Conclusion

Collectively, these findings highlight the complexity of *Alternaria* leaf blight (ALB) in mustard, which is caused by multiple Alternaria species varying in virulence and genetic makeup. The combined use of morphological, pathogenicity, and molecular tools provided a comprehensive framework for accurate species identification and offered insights into the genetic potential for toxin biosynthesis in *A*. *alternata*. Importantly, the presence of highly virulent *A. alternata isolates* carrying conserved polyketide synthase genes associated with toxin biosynthesis underscores the need for resistance breeding programs targeting toxin-associated pathogenicity, the identification of species-specific resistant donors, and region-specific disease management strategies. Future research should focus on functional validation of the pksH and pksJ genes, their expression during host infection, and quantitative analysis of Alternaria toxins to better understand their role in host–pathogen interactions, which may open new avenues for molecular breeding and biocontrol interventions.

## Data Availability

The datasets presented in this study can be found in online repositories. The names of the repository/repositories and accession number(s) can be found in the article/supplementary material.
